# Bristol University: Department of Preventive Medicine
*Reprinted by kind permission of the Editor from the *British Medical Journal*, 7th October, 1933.


**Published:** 1933

**Authors:** 


### BRISTOL UNIVERSITY: DEPARTMENT OF PREVENTIVE MEDICINE.*

### OPENING BY THE MINISTER OF HEALTH.

On Friday, 29th September, the Minister of Health, Sir Hilton Young, formally opened a new Department of Preventive Medicine at the University of Bristol. The Department is housed in Canynge Hall, which has been adapted and equipped for the purpose. Under an agreement between the University and the city the bacteriology, pathology, and preventive medicine work of Bristol and its municipal hospitals will be carried out by this Department, which will receive a grant from the city for its maintenance. In order to facilitate arrangements Professor Walker Hall resigned his Professorship of Pathology and was appointed Director of the new laboratories, and Dr. R. H. Parry was appointed Honorary Professor of Preventive Medicine in the University. The vacant Chair of Pathology has been filled by the appointment of Professor G. Hadfield of the Royal Free Hospital (formerly Pathologist to the Bristol General Hospital), who will devote himself to research and teaching. The promised co-operation of the pathologists at the Royal Infirmary and the General Hospital will enable Professor Hadfield to draw his material and give instruction to his classes in those two institutions.

* Reprinted by kind permission of the Editor from the *British Medical Journal*, 7th October, 1933.

The association of the Preventive Medicine Department with the municipal hospitals at Southmead, Ham Green, Frenchay and elsewhere will likewise serve to increase the material to which medical students and research workers in Bristol will have access. The Professors of Medicine, Surgery and Obstetrics are also accommodated in Canynge Hall, so that for the present all academic lectures in the clinical subjects will be given there.

At the inauguration ceremony the Vice-Chancellor, Dr. Thomas Loveday, welcomed the Minister of Health in the large Lecture Theatre before a considerable assembly of guests, including the Lord Mayor of Bristol, the Sheriff (Dr. Kenneth Wills), Alderman Maggs (Chairman of the Health Committee), Dr. Stanley Badock (Chairman of the University Council), and Mr. Hiatt Baker (Pro-Chancellor).

Dr. Loveday described the steps by which the connection between the City Health Services and the University Pathological Department had grown up during more than thirty years, until it had culminated in this complete union in the Department of Preventive Medicine. It was, he said, a difficult experiment to start, but it would be easy to carry on when well started. Both parties to the agreement expected that it would work happily under the direction of Dr. Walker Hall, with the Medical Officer of Health, Dr. Parry, in the Chair of Preventive Medicine.

Alderman Maggs, as Chairman of the Health Committee, said he looked upon this development as a great step forward in the interests of the public health for the protection of food supplies, for the earlier recognition of disease and for its control and treatment.

Sir Hilton Young, declaring the new Department open, referred to the tour of Bristol he had just made—viewing the new housing estates, the city's efforts at slum clearance, the homes for children, the nursery school, the municipal hospitals, particularly the Orthopædic Hospital at Frenchay and Hortham Colony for Mental Defectives. He congratulated the city on feeling forward along the lines of development of the public health services and grasping at new opportunities. Bristol had its slum problem, a legacy of old days with lower standards, but it had also its legacy of traditional progress. "This Department of Preventive Medicine will stand as an example of vigour and foresight adequate to your needs. It should give to everyone encouragement and high hope to continue the efforts of a great and honourable past."

Dr. Walker Hall and Dr. Parry also spoke, each paying tribute to the great preventive work carried out by the late Dr. D. S. Davies, who for many years was Medical Officer of Health for Bristol.

Dr. S. Badock proposed a vote of thanks to Sir Hilton and Lady Young, and referring to Sir Hilton Young's remark that the present celebration was a marriage of the City of Bristol with the fair nymph of knowledge, said that it was a most modern type of marriage, in that it was subject to revision from time to time, but this provision would probably achieve a lasting and happy union.

After the opening ceremony the Minister of Health was the guest of the University at lunch in the University Union. The Lord Mayor (Councillor Wise), in the course of his speech proposing the health of Sir Hilton and Lady Young, mentioned with pride that Bristol possessed an example of Lady Young's artistic handiwork in the Cathedral.

Sir Hilton Young, replying, said that he would not have asked for this opportunity of paying Bristol a visit if he had not been confident that he would find there a discharge of the services for which he was responsible that was in the best standard of national achievement. "I can confirm from my personal impressions and knowledge," he said, "that you need not fear comparison with other great cities of the country."

Dr. Middleton Martin, Medical Officer of Health for Gloucestershire, spoke of the value to the county in the past of the University's Pathological Department, and welcomed this extension as promising to be of even greater service in the future. He, like other speakers, referred to the splendid work of the late Dr. D. S. Davies.

Undoubtedly the occasion marks a further stage in the closer co-operation between the State and voluntary organizations which is all to the good. It is perhaps singular that throughout the proceedings no speaker mentioned Bristol's greatest contribution to preventive medicine in the discovery by Dr. William Budd of the mode of transmission of typhoid fever. Dr. Budd's rules for nursing typhoid cases are to this day employed by the whole civilized world.

